# Efficacy after preoperative capecitabine and oxaliplatin (XELOX) versus docetaxel, oxaliplatin and S1 (DOS) in patients with locally advanced gastric adenocarcinoma: a propensity score matching analysis

**DOI:** 10.1186/s12885-018-4615-z

**Published:** 2018-06-28

**Authors:** Yan Wang, Xi Cheng, Yue-hong Cui, Jun Hou, Yuan Ji, Yi-hong Sun, Zhen-bin Shen, Feng-lin Liu, Tian-shu Liu

**Affiliations:** 1 Department of Medical Oncology, Zhongshan Hospital, Fudan University, Shanghai, China; 2 Department of Pathology, Fudan University, Zhongshan Hospital, Shanghai, China; 3 Department of General Surgery, Fudan University, Zhongshan Hospital, Shanghai, China; 4 Center of Evidence-based Medicine, Fudan University, Shanghai, China; 5 Fudan University, ZhongShan Hospital, 180 Fenglin Road, Shanghai, 200032 People’s Republic of China

**Keywords:** Locally advanced gastric cancer, Preoperative chemotherapy, Curative resection rate, Propensity score matching analysis

## Abstract

**Background:**

The aim of this study was to compare the efficacies of the XELOX and DOS regimens as preoperative chemotherapy in patients with locally advanced gastric cancer.

**Methods:**

All cases of locally advanced gastric cancer treated with the XELOX or DOS regimen were reviewed retrospectively. Propensity score matching (PSM) was carried out to reduce selection bias based on age, gender, location, Lauren type, carcinoembryonic antigen level, clinical tumor stage, and clinical node stage.

**Results:**

From January 2010 to December 2016, 248 patients were matched; 159 of them received the XELOX regimen and 89 the DOS regimen. The response rates in the XELOX and DOS groups were 34.5 and 38.1%, respectively (*P* = 0.823). After four cycles of chemotherapy, 111 patients (69.8%) in the XELOX group and 65 patients (73.0%) in the DOS group underwent radical surgery (*P* = 0.485). The median progression-free survival (33.0 months vs. 18.7 months, *P* = 0.0356) and the median overall survival (43.8 months vs. 29.1 months, *P* = 0.0003) were longer for patients who received the DOS regimen than for those who received the XELOX regimen. The occurrence of grade 3 to 4 toxicity was similar in the two groups.

**Conclusions:**

For locally advanced gastric cancer patients, the DOS regimen showed more benefit than the XELOX regimen as preoperative chemotherapy, without any added toxicity effects.

## Background

Gastric cancer is the third leading cause of cancer-related death in both sexes worldwide, and half of new cases occur in Eastern Asia, mainly in China [1]. Surgical resection is the curative treatment for early stage gastric cancer [2]. However, gastric cancer patients are generally diagnosed at an advanced stage with extensive regional nodal involvement or invasion of adjacent structures. In China, the 5-year overall survival (OS) has ranged from 29 to 53% for stage III gastric cancer [3]. Some investigators have reported that preoperative chemotherapy can reduce the tumor burden and enhance the probability that patients can be treated with radical resection [4, 5]. The MAGIC trial showed that three cycles of epirubicin, cisplatin and continuous 5-fluorouracil (5-Fu) infusion (ECF) before and after surgery led to a significant improvement in OS in advanced gastroesophageal adenocarcinoma in western countries [6]. However, although the response rate to the triplet combination could be high, severe toxicities and chemotherapy intolerance after operation may limit its use in clinical practice. Also, the MAGIC trial showed that only 43% percent of patients completed all six cycles of the protocol regimen and 34% percent of patients did not begin postoperative chemotherapy after surgery [6]. In a previous study, our group showed that the efficacy of combinations of fluoropyrimidines and oxaliplatin was similar with or without the inclusion of an anthracycine. Furthermore, the side effects tended to occur more frequently with the addition of an anthracycline, especially leucopenia, fatigue and vomiting [7].

Recently, docetaxel has be shown to be effective in locally advanced gastric cancer within the preoperative setting. The addition of docetaxel to cisplatin or oxaliplatin and fluorouracil has been used increasingly more often to improve outcomes among these patients [8–12]. The responses to these docetaxel-based triplet combinations exceeded 50% after two or three cycles of preoperative chemotherapy, which was better than outcomes reported for anthracycline-based triplet regimens. The rate of a complete pathological response to the docetaxel-based regimen also was higher in patients with resectable disease (e.g., 16% vs. 6% after four cycles of an anthracycline-based regimen in the FLOT study). Regarding the safety of the regimen, preoperative treatment with docetaxel-based combinations for locally advanced gastric cancer demonstrated sufficient clinical efficacy with manageable toxicities.

In our experience, doublet regimens such as the XELOX regimen (capecitabine plus oxaliplatin) can induce a more favorable tumor response rate with a relatively mild toxicity profile for locally advanced gastric cancer patients compared with the EOX (epirubicin, cisplatin and capecitabine) regimen [7]. However, since it is unknown whether the docetaxel-based triplet combination produces better clinical effects compared to the XELOX regimen for enabling curative resection, the objective of this study was to determine which chemotherapy regimen (the DOS or XELOX regimen) can make subsequent radical surgery feasible and improve OS in patients with locally advanced gastric cancer.

## Methods

### Patient selection

This study retrospectively reviewed consecutive cases of locally advanced gastric cancer who were prospectively registered at our institution and treated with the XELOX or DOS regimen as preoperative chemotherapy. Data were obtained from databases maintained by the Department of Medical Oncology of Zhongshan Hospital, Fudan University. Patients were eligible if they had histologically confirmed adenocarcinoma of the stomach or EGJ (esophagogastric junction) and were regarded as having clinical stage ≥cT3 and nodal positive (cN+) disease as assessed by endoscopic ultrasound examination, contrast computed tomography (CT) scanning of the chest, abdomen, and pelvis. The protocol for this trial was approved by the institutional ethical board of Zhongshan Hospital, Fudan University and was registered on ClinicalTrials.gov (NCT02623153).

### Preoperative chemotherapy

Patients received either the XELOX or DOS regimen according to the physician’s preference. In the XELOX regimen, capecitabine was provided at 1000 mg/m^2^, twice a day on days 1–14 and oxaliplatin was provided at 130 mg/m^2^ on day 1. The DOS regimen was planned as S-1 (Tegafur, Gimeracil and Oteracil Porassium Capsules) of 40 mg/m^2^ orally administered twice a day on days 1–14, oxaliplatin at 100 mg/m^2^, and docetaxel at 40 mg/m^2^ on day 1. The two regimens were repeated every 3 weeks.

### Surgery and pathological evaluation

After two cycles (6 weeks) of chemotherapy, CT scan of the chest and the whole abdomen were carried out to evaluate the tumor response. Surgical resection was performed within 4–6 weeks after four cycles of treatment. Radical resection was aimed for by gastrectomy with an extended lymph node resection (D2). Postoperative adjuvant chemotherapy consisted of previous regimen for four cycles after curative resection. Assessment of response was evaluated according to the response evaluation criteria in solid tumor (RECIST) 1.1 [13] and for primary lesions according to the guidelines of the Japanese classification of gastric carcinoma [14]. Toxicity and adverse events were reported using the Common Toxicity Criteria of the National Cancer Institute (NCI –CTC) 3.0 [15]. All resected specimens were examined by pathologists and tumor regression grade to chemotherapy was quantified according to the Japanese classification of gastric carcinoma regression criteria [16].

### Statistical analysis

The primary study endpoint of this trial was the response rate. Secondary endpoints were curative resection rate, progression-free survival (PFS), OS, and toxicity. The categorical parameters were compared using chi-square test. Kaplan–Meier method techniques utilized to estimate the PFS and OS and significant differences in survival curves between comparative groups were compared by the log-rank test. Propensity score matching (PSM) was performed to reduce selection bias between patients treated with the XELOX and DOS regimens. PSM accounted for factors of age, gender, location, Lauren type, carcinoembryonic antigen (CEA) level, clinical Tumor (T) stage, and clinical node (N) stage. Propensity scores were calculated using a logistic regression model and a nearest neighbor matching algorithm with a matching ratio of 1:2. The balance of covariates was assessed according to their standardized differences. A difference < 10% of the absolute value was considered significantly balanced. SPSS software (version 22.0; SPSS, Chicago, IL) was used for statistical analyses. *P* < 0.05 was considered significant.

## Results

### Baseline characteristics

**Fig. 1:**
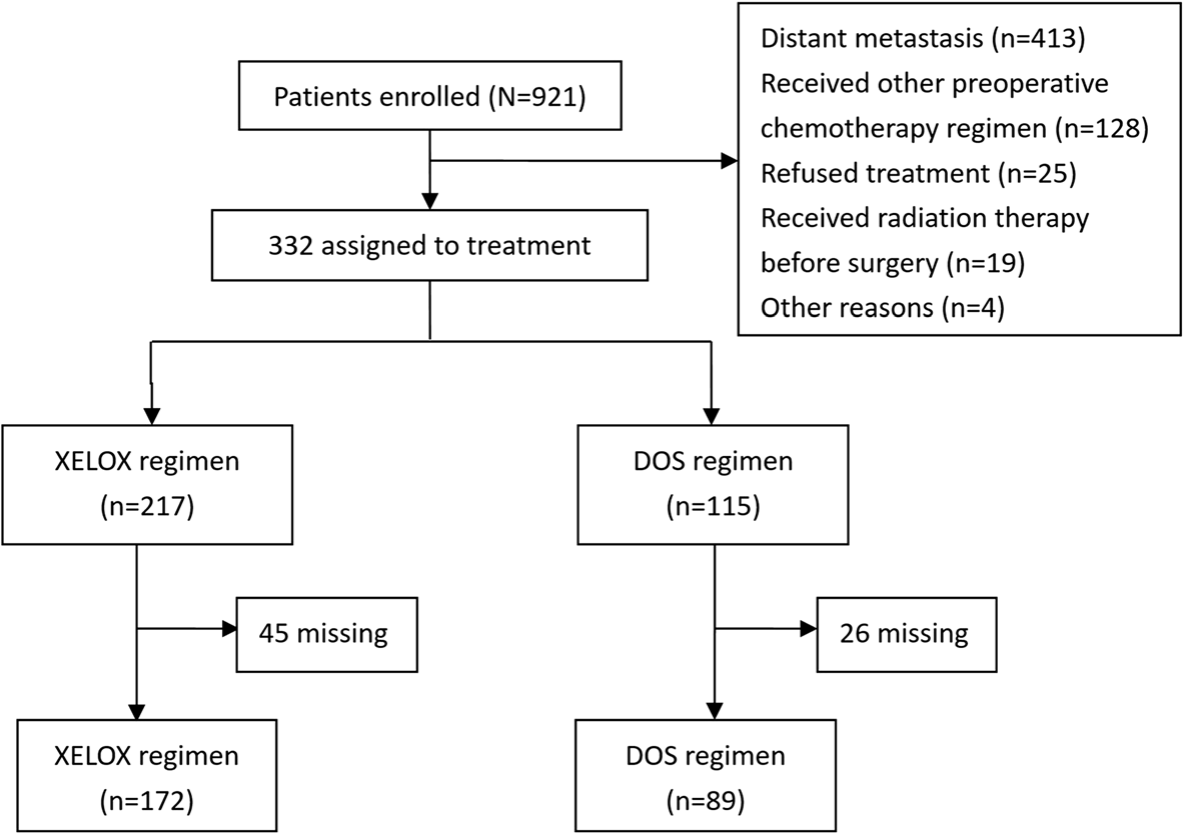
Consort diagram of the study

Between January 2010 and December 2016, 921 patients were enrolled in the study. After screening, 589 patients were excluded due to the existence of distant metastasis, having received another preoperative chemotherapy regimen, refusal of treatment, or other reasons. Then 71 patients missed follow-up within the first two treatment cycles. Thus, the study population included 248 patients, among whom 159 received the XELOX regimen and 89 received the DOS regimen (Fig. 1). All patients were followed up for comprehensive data collection, and the last date of follow-up was August 31, 2017. The baseline characteristics of the patients before and after PSM are shown in Table 1. After adjustment of background factors by PSM, the two groups were well balanced with respect to gender, age, gender, location, Lauren type, CEA level, and clinical stage.

**Table 1:** Comparison of characteristics before and after propensity score matching

Variables	Before matching			After matching		
	XELOX group (*n* = 172)	DOS group (*n* = 89)	*P*	XELOX group (*n* = 159)	DOS group (*n* = 89)	*P*
Gender			0.130			0.224
Male	131 (76.1)	60 (67.4)		120 (75.5)	60 (67.4)	
Female	41 (23.9)	29 (32.6)		39 (24.5)	29 (32.6)	
Age (median year, range)			0.152			0.214
Median	62	59		62	59	
Range	31–78	24–74		31–78	24–74	
⩾ 60	101 (58.7)	44 (49.4)		93 (58.5)	44(51.2)	
Location			0.857			0.966
Gastroesophageal junction	56 (32.5)	28 (31.5)		51 (32.1)	28 (31.5)	
Stomach	116 (67.5)	61 (68.5)		108 (67.9)	61 (68.5)	
Lauren type			0.043			0.167
Intestinal type	96 (55.8)	42 (47.2)		89 (55.9)	42 (47.2)	
Diffuse type	48 (27.9)	38 (44.7)		49 (29.0)	38 (44.7)	
Mixed type	28 (16.3)	9 (8.1)		21 (15.1)	9 (8.1)	
CEA			0.055			0.197
Normal	133 (77.3)	59 (66.3)		119 (74.8)	59 (66.3)	
Elevated	39 (22.7)	30 (33.7)		40 (25.2)	30 (33.7)	
Causes of unresection			0.493			0.454
T4b	11 (6.4)	7 (7.8)		10 (6.3)	7 (7.8)	
Borrmanntype 4 or large type 3	57 (33.1)	35 (39.3)		52 (32.7)	35 (39.3)	
Bulky lymph nodes	104 (60.5)	47 (52.9)		97 (64.0)	47 (52.9)	
Clinical T stage			0.579			0.535
cT3	9 (5.2)	7 (7.9)		8 (5.0)	7 (7.9)	
cT4	163 (94.8)	82 (92.1)		151 (95.0)	82 (92.1)	
Clinical N stage			0.485			0.459
cN1	56 (32.6)	32 (36.0)		52 (32.7)	32 (36.0)	
cN2	73 (42.4)	31 (34.8)		68 (42.8)	31 (34.8)	
cN3	43 (25.0)	26 (29.2)		39 (24.5)	26 (29.2)	

### Response to the chemotherapy (Table 2)

Preoperative chemotherapy data were available for all 248 patients. After four cycles, only 245 cases could be evaluated for response as three patients experienced acute stomach perforation after the first cycle of the XELOX regimen and thus did not receive the evaluation. The results showed no differences in outcomes between the two groups. In the XELOX group, two patients had a complete response (CR), 53 had partial responses (PR), 87 had stable disease (SD), and 14 had progression of disease (PD). The response rate (RR) was 34.5%, and disease control rate (DCR) was 89.2%. In the DOS group, the RR and DCR were 38.1 and 89.7%, respectively, which suggested no significant difference in the response rates between the two groups.

**Table 2:** Response of neoadjuvant chemotherapy and surgery resection rate in the two groups (*N* = 248)

	XELOX group (*N* = 159)	DOS group (*N* = 89)	*P*
Response evaluation			0.823
CR	2 (1.2)	1 (1.1)	
PR	53 (33.3)	33 (37.0)	
SD	87 (54.7)	46 (51.6)	
PD	14 (8.9)	9 (10.3)	
Not assessable	3* (1.9)	0 (0)	
RR (CR plus PR)	55 (34.5)	34 (38.1)	0.569
DCR (CR plus RR plus SD)	142 (89.2)	80 (89.7)	0.886
Patients received surgery			0.4850
Radical surgery	111 (69.8)	65 (73.0)	
Non-radical surgery	48 (30.2)	34 (27.0)	

*There patients in XELOX group did not have response evaluation because of acute stomach perforation after the first regimen of chemotherapy

### Surgical findings and pathology staging (Table 3)

Ultimately, 111 patients in the XELOX group and 65 in the DOS group were able to undergo radical resection. The rate of resection with curative intent was similar between the two groups (69.8% vs. 73.0%, *P* = 0.485). The results for pathological regression are shown in Table 3. Among patients who underwent radical operation, a significantly higher proportion of patients achieved a pathological response in the DOS group than in the XELOX group (48 of 65 patients [73.8%] in the DOS group vs 59 of 111 patients [53.1%] in the XELOX group; *P* = 0.01). The proportion of patients who achieved pathological CR was similar in the two groups (9.0% vs. 12.9%, *P* = 0.685). The median number of dissected lymph nodes (30 vs. 34) was close in both groups. The median number of positive lymph nodes also showed no difference between the two groups (4 vs. 2, respectively). The median time from surgery to discharge was 11 days (range, 6–39 days) in the XELOX group and 13 days (range, 5–43 days) in the DOS group. On postoperative staging, the proportion of patients in the DOS group with a low pathological tumor stage (< ypT4) was greater than that in the XELOX group (*P* = 0.038).

**Table 3:** Surgical findings for the patients received radical surgery after chemotherapy (*N* = 176)

	XELOX group (*n* = 111)	DOS group (*n* = 65)	*P*
Pathological response			
Responders	59 (53.1)	48 (73.8)	0.010
pCR	10 (9.0)	8 (12.9)	0.685
Median total nodes	30 (2–75)	34 (9–71)	
Median positive nodes	4 (0–62)	2 (0–30)	
Median time from end of treatment to surgery	29 (16–38)	30 (17–42)	
Median time from surgery to discharge	11 (6–39)	13 (5–43)	
Pathological T stage			
ypT0	13 (11.7)	9 (13.8)	0.623
ypT1	14 (12.6)	7 (10.7)	0.715
ypT2	13 (11.7)	12 (18.5)	0.215
ypT3	35 (31.5)	26 (40.0)	0.254
ypT4	36 (32.5)	11 (17.0)	0.024
Combined ypT0/ypT1/ypT2/ypT3	75 (67.5)	54 (83.0)	0.038
Pathological N stage			
ypN0 (no regional lymph nodes)	35 (31.5)	26 (40.0)	0.329
ypN1 (1–2 positive lymph nodes)	17 (15.3)	12 (18.5)	0.739
ypN2 (3–6 positive lymph nodes)	23 (20.7)	10 (16.1)	0.499
ypN3 (> 6 positive lymph nodes)	36 (32.5)	17 (28.4)	0.480

### Survival

**Fig. 2:**
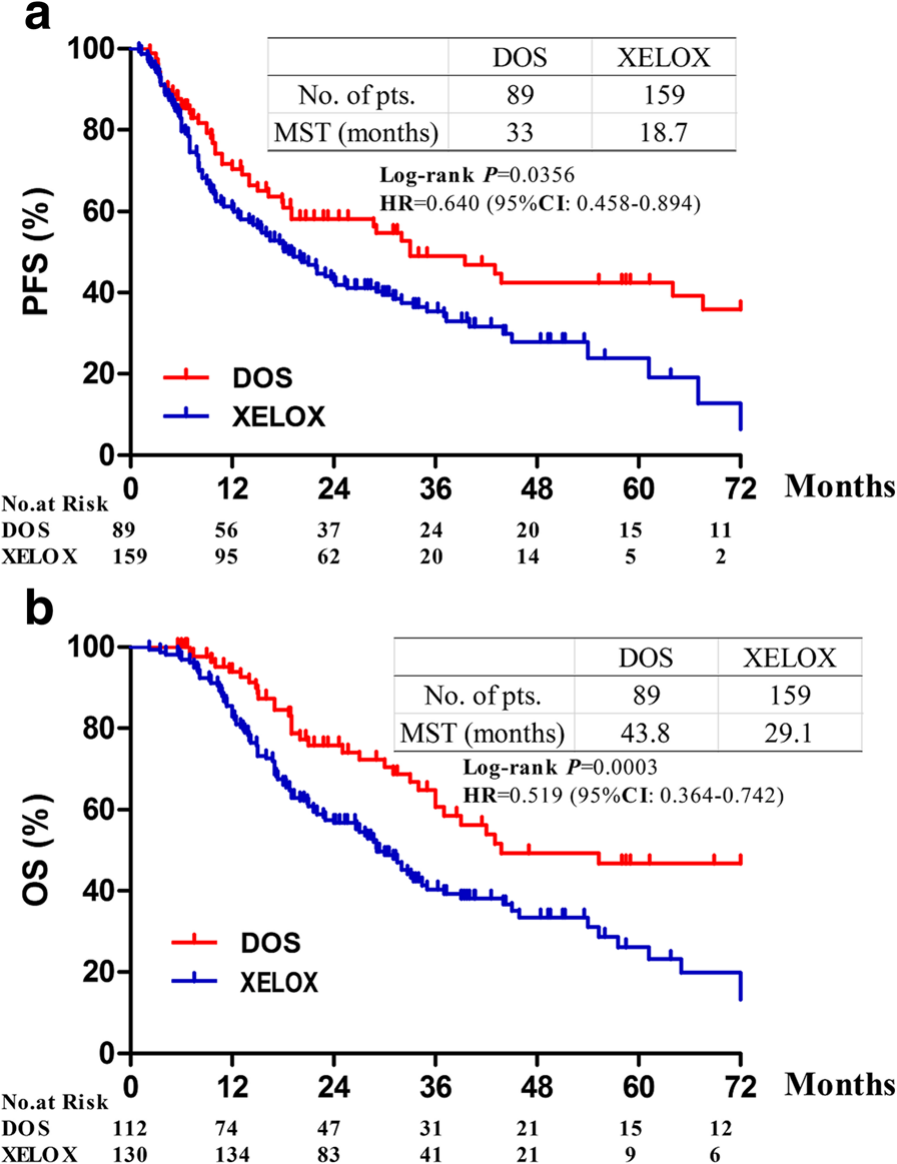
Progression-free and overall survival according to treatment. **a** Progression-free survival. **b** Overall survival. HR = hazard ratio

After a median follow-up of 18.7 months (range, 2.2–87.3 months), 151 patients (106 in the XELOX group and 45 in the DOS group) experienced disease progression or relapse, and 133 patients (99 in the XELOX group and 34 in the DOS group) had died. The median PFS was 18.7 months in the XELOX group and 33.0 months in the DOS group (hazard ratio [HR] 0.64; 95% confidence interval [CI] 0.458–0.894, *P* = 0.0356). The median OS was 29.1 months in the XELOX group and 43.8 months in the DOS group (HR 0.519; 95% CI 0.364–0.742, *P* = 0.0003, Fig. 2).

**Fig. 3:**
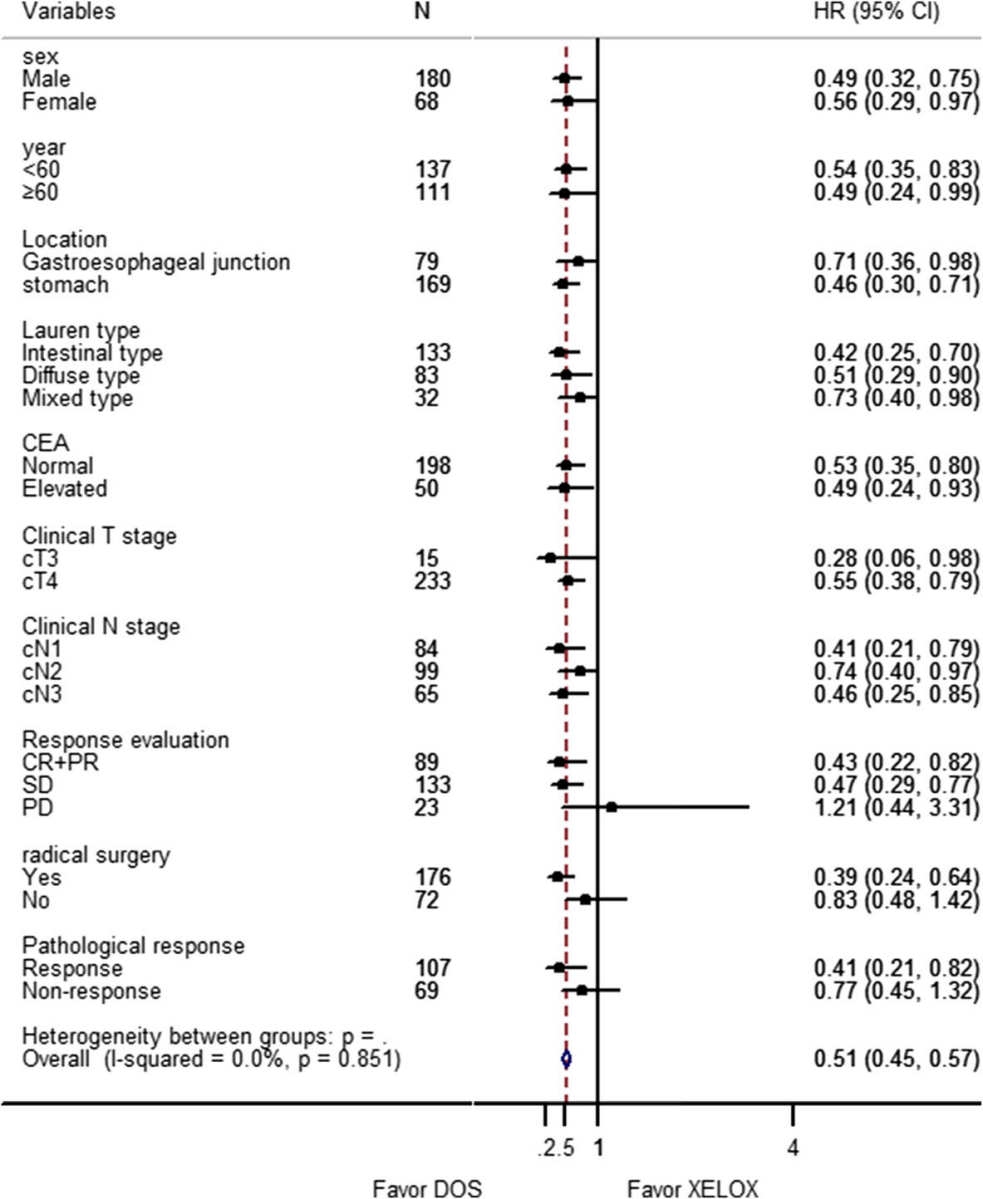
Forest plot of the treatment effect on overall survival in subgroup analysis

**Fig. 4:**
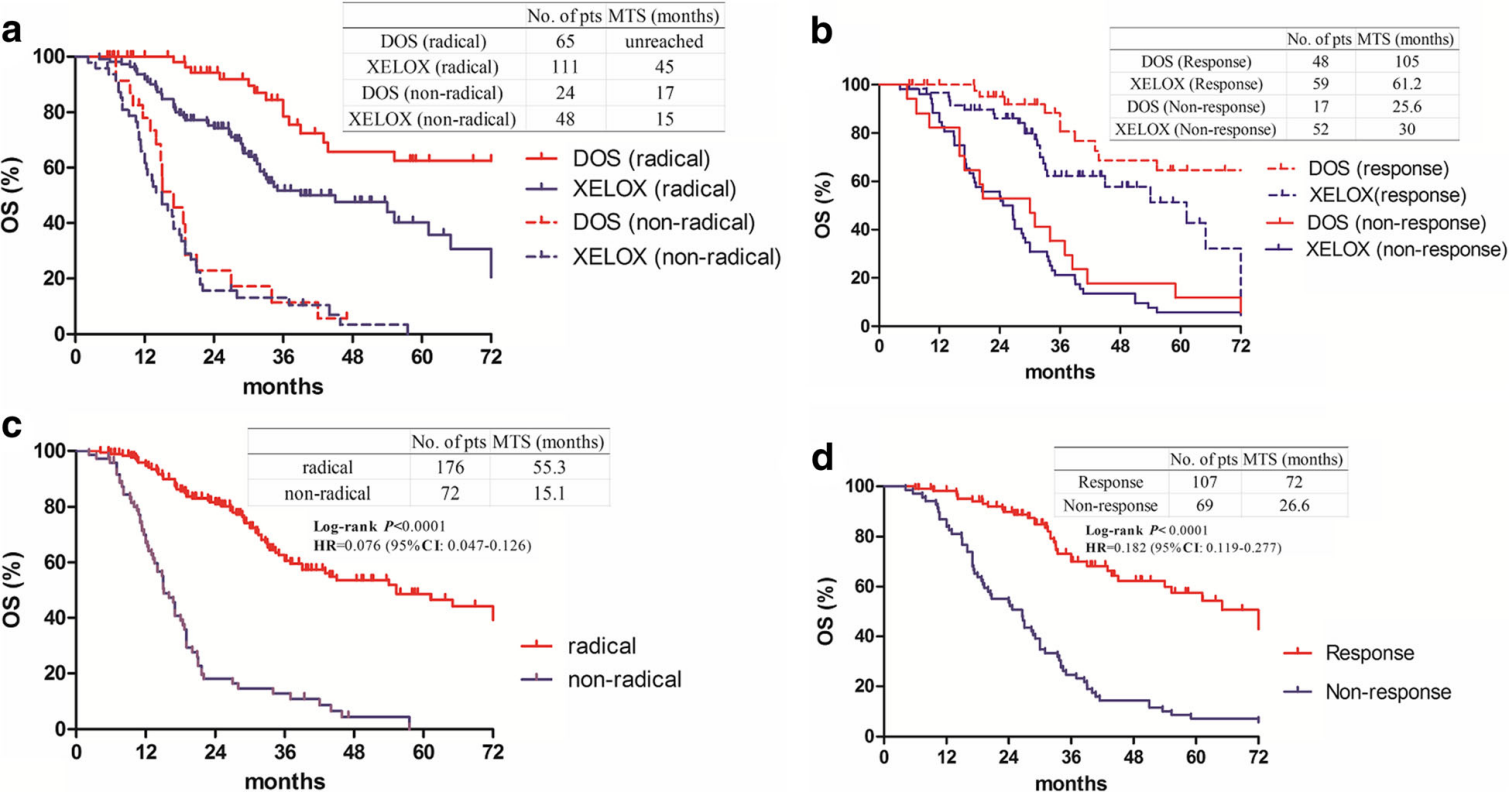
Overall survival according to resection and pathological response. **a** Overall survival in patients treated with or without radical resection between the DOS and XELOX groups. **b** Overall survival in responders or non-responders between the DOS and XELOX groups. **c** Overall survival in patients treated with or without radical resection. **d** Overall survival in pathological responders or non-responders

The results of subgroup analysis are shown in Fig. 3. The effect of DOS compared with XELOX on OS was significantly favorable in variables of sex, age, location, Lauren type, CEA level, clinical T stage and clinical N stage. There was no statistical benefit of either regimen in patients with disease progression (HR 1.21, 95% CI 0.44–3.31, *P* = 0.769), without radical surgery (HR 0.83, 95% CI 0.48–1.42, *P* = 0.298), or without a pathological response (HR 0.77, 95% CI 0.45–1.32, *P* = 0.637). Patients who underwent radical resection had a better OS in the DOS group than in the XELOX group, as did those who had a pathological response (Fig. 4a and b). Patients who underwent a radical resection had a significantly longer median OS of 55.3 months than patients not treated with radical resection (15.1 months, HR 0.076, 95% CI 0.047–0.126, *P* < 0.0001, Fig. 4c). Resection followed response to chemotherapy, and tumor response itself had a major influence on the OS (HR 0.182; 95% CI 0.119–0.277, *P* < 0.0001, Fig. 4d).

### Toxicity (Table 4)

Overall, the toxicity observed was mostly mild in both groups, and no deaths were attributable to chemotherapy or surgery. The most common adverse events were leukocytopenia and thrombocytopenia. The two groups experienced similar serious adverse effects in bone marrow and other toxicities.

**Table 4:** Grade 3/4 events in the whole population (*N* = 248)

Toxicities	XELOX group (*N* = 159)	DOS group (*N* = 89)	*P*
leukocytopenia	6 (3.7)	3 (3.4)	0.848
Febrile neutropenia	1 (0.6)	0 (0)	0.768
thrombocytopenia	5 (3.1)	3 (3.4)	0.781
anemia	2 (1.3)	0 (0)	0.747
nausea	2 (1.3)	1 (1.1)	0.608
vomiting	1 (0.6)	1 (1.1)	0.747
diarrhea	1 (0.6)	0 (0)	0.768
hand-foot skin reaction	3 (1.9)	1 (1.1)	0.945
hepatic dysfunction	2 (1.3)	1 (1.1)	0.608
neuropathy	2 (1.3)	0 (0)	0.747
Mucositis	3 (1.9)	1 (1.1)	0.946

## Discussion

In this study of preoperative chemotherapy in Chinese patients with locally advanced gastric cancer, we first demonstrated the benefits of the additional use of docetaxel to fluoropyrimidines and oxaliplatin as compared with the XELOX regimen alone. We confirmed that when docetaxel was added, the DOS regimen could lead to a greater rate of pathological response and improved survival benefit. The optimal regimen for preoperative chemotherapy in locally advanced gastric cancer is still unclear. Combinations of fluoropyrimidines and platinum drugs with or without an anthracycline have been the most frequently tested regimens. Unfortunately, our previous study demonstrated that adding an anthracycline to this combination did not show advantages in terms of response and survival. Nowadays, as increasing numbers of new chemotherapy agents become available such as docetaxel and paclitaxel, which have demonstrated promising efficacy and manageable toxicity [8–11], new triplet regimens including more powerful agents should be considered. Lorenzen et al. [17] demonstrated that additional docetaxel to infusional 5-FU and oxaliplatin (FLOT) as neoadjuvant chemotherapy offers a better chance for radical resection compared with FLO (infusional 5-FU and oxaliplatin) in elderly locally advanced gastroesophageal cancer patients. Compared with the FLO group, the FLOT group showed a trend towards an improved median PFS (21.1 vs 12.0 months; *P* = 0.09). Similarly, we also demonstrated a survival benefit with the use of additional docetaxel as compared with fluoropyrimidines and platinum alone, with estimated improvements in PFS of 14.3 months and in OS of 14.7 month.

The results for pathological response are shown in Table 3. A significantly high proportion of patients achieved a pathological regression after treatment with the DOS regimen than after treatment with the XELOX regimen (73.8% vs 53.1, *P* = 0.01), which was consistent with other reported studies [8–11]. Likewise, the pathological responses to docetaxel-based triplets could reach from 65.9–71.2% in previous small single-center studies. However, the difference in the clinical response rate between the two groups did not correspond to the rate of histopathological regression. In the population after PSM, clinical response was achieved in 34 (38.1%) of 89 patients in the DOS group and 55 (34.5%) of 156 patients in the XELOX group (*P* = 0.569). The Phase II COMPASS study [18] evaluated the accuracy of radiological diagnosis after neoadjuvant chemotherapy in 75 patients and showed that the accuracy and sensitivity of restaging after preoperative chemotherapy was inferior compared with primary staging. That is because the metabolic changes induced by chemotherapy often precede anatomical changes by which overdiagnosis can often occur especially in responders. On the contrary, pathological evaluation has higher accuracy compared with radiological diagnosis after preoperative chemotherapy.

In this study, we demonstrated that patients who received the DOS regimen achieved a better median OS (43.8 months vs. 29.1 months, *P* = 0.0003) and a better median PFS (33.0 months vs. 18.7 months, *P* = 0.0356) than those who received the XELOX regimen. We also confirmed the survival benefits of radical resection and pathological response to preoperative chemotherapy, consistent with the results of previous studies [12, 19]. The median OS could be prolonged from 15.1 months to 55.3 months when preoperative chemotherapy and radical surgery were sequentially accomplished (Fig. 4); meanwhile, the median OS of patients with a pathological response could be extended from 26.6 months to 72 months as well. In patients with resectable gastric cancer, we demonstrated a survival benefit with the DOS regimen as compared with the XELOX regimen. However, there was no difference in survival between the groups among patients who did not undergo radical surgery. The results showed that gastric cancer patients may not benefit from docetaxel-based triplet combinations as palliative chemotherapy without radical resection. Similarly, Al-Batran et al. evaluated the feasibility of triple- versus double-drug chemotherapy in elderly patients with esophagogastric cancer [20]. The triplet combination improved PFS in the subgroup with locally advanced disease (10.3 months vs. 24.2 months, *P* = 0.019), but not in the group with metastasis (6.0 months vs. 7.3 months, *P* = 0.43).

Additionally, it has been traditionally thought that more adverse effects will occur in the more aggressive therapy containing triplet combinations. Our data indicated that the side effects of preoperative chemotherapy were similar in the DOS group and the XELOX group. Similar studies including pooled review, which compared chemotherapy with docetaxel versus non-docetaxel-containing regimens, showed that docetaxel-containing three-drug regimens have increased response rates, but the advantages of the docetaxel-containing three-drug combinations are counterbalanced by increased toxicity [12, 20, 21]. However, as the reported docetaxel-combined triplet combinations are administered every 2 weeks and intravenous flurouracil was included rather than an oral agent in our study, there was a difference in the side effects between our new triplet combination and other docetaxel-containing three-drug combinations. Meanwhile, the dose of oxaliplatin was higher in the XELOX regimen than in the DOS regimen, which could partially explain the identical side effects between these doublet and triplet regimens.

Some limitations of the present study must be acknowledged. First, this was a retrospective study performed in a single institution and patients received either the XELOX or DOS regimen according to the physician’s preference which corresponded to a non-randomized, unblinded setting with a high risk of selection bias. Second, we did not routinely perform laparoscopic exploration for the patients with locally advanced gastric cancer, which is regarded as the most helpful procedure to detect peritoneal dissemination with high sensitivity and specificity. Additionally, although we used propensity score matching to balance the selection bias, some unrecorded potential confounders, such as performance status and comorbid disease, were not included in the study.

## Conclusion

In conclusion, our study suggested that additional of docetaxel to fluoropyrimidines and a platinum compound is an effective treatment option as a preoperative chemotherapy regimen for locally advanced gastric cancer patients. Meanwhile, our data strengthen the evidence that patients can tolerate an aggressive multimodal treatment approach with a docetaxel-based triple-drug therapy, followed by radical surgery. Further large-scale randomized investigations focused on certain types of regimen are therefore required to validate the best strategy.

## Abbreviations

5Fu: 5-fluorouracil
CEA: Carcinoembryonic antigen
CR: Complete response
CT: Computed tomography
DCR: Disease control rate
DOS: Docetaxel, oxaliplatin and S1
ECF: Epirubicin, cisplatin and continuous 5-fluorouracil infusion
EGJ: Esophagogastric junction
EOX: Epirubicin, cisplatin and capecitabine
FLO: Infusional 5-FU, leucovorin, and oxaliplatin
FLOT: Infusional 5-FU, leucovorin, oxaliplatin and docetaxel
N: Clinical node
NCI –CTC: Common Toxicity Criteria of the National Cancer Institute
OS: Overall survival
pCR: Pathologic complete response
PD: Progression of disease
PFS: Progression-free survival
PR: Partial response
PSM: Propensity score matching
RECIST: Response evaluation criteria
RR: Response rate
S-1: Tegafur, Gimeracil and Oteracil Porassium Capsules
SD: Stable disease
T: Clinical tumor
XELOX: Capecitabine and oxaliplatin
